# Impact on sexual function of surgical treatment in rectal cancer

**DOI:** 10.1590/S1677-5538.IBJU.2017.0318

**Published:** 2018

**Authors:** Pedro Costa, João M. Cardoso, Hugo Louro, Jorge Dias, Luís Costa, Raquel Rodrigues, Paulo Espiridião, Jorge Maciel, Luís Ferraz

**Affiliations:** 1 Departmento de Urologia, Centro Hospitalar Vila Nova de Gaia, Espinho, Portugal;; 2 Departmento de Cirurgia Geral, Centro Hospitalar Vila Nova de Gaia, Espinho, Portugal

**Keywords:** Surgical Procedures, Operative, Rectal Neoplasms, Diagnosis

## Abstract

**Introduction:**

The development of new surgical techniques and medical devices, like therapeutical multimodal approaches has allowed for better outcomes on patients with rectal cancer (RCa). Owing to that, an increased awareness and investment towards better outcomes regarding patients’ sexual and urinary function has been recently observed.

**Aim:**

Evaluate and characterize the sexual dysfunction of patients submitted to surgical treatment for RCa.

**Materials and Methods:**

An observational retrospective study including all male patients who underwent a surgical treatment for RCa between January 2011 December 2014 (n=43) was performed, complemented with an inquiry questionnaire to every patient about its sexual habits and level of function before and after surgery.

**Discussion:**

All patients were male, with an average of 64yo. (range 42-83yo.). The surgical procedure was a rectum anterior resection (RAR) in 22 patients (56%) and an abdominoperineal resection (APR) in 19(44%). Sixty three percent described their sexual life as important/very important. Sexual function worsening was observed in 76% (65% with complains on erectile function, and 27% on ejaculation). Fourteen patients (38%) didn't resume sexual activity after surgery. Increased age (p=0.007), surgery performed (APR) (p=0.03) and the presence of a stoma (p=0.03) were predictors of ED after surgery. A secondary analysis found that the type of surgery (APR) (p=0.04), lower third tumor's location (p=0.03) and presence of comorbidities (p=0.013) (namely, smokers and diabetic patients) were predictors of *de novo* ED after surgery.

**Conclusions:**

This study demonstrated the clear negative impact in sexual function of patients submitted to a surgical treatment for RCa. Since it is a valued feature for patients, it becomes essential to correctly evaluate/identify these cases in order to offer an adequate therapeutical option.

## INTRODUCTION

Colorectal cancer is one of world's most frequent neoplasms, ranked in third in Portugal (1). Almost 10% of cancer survivors underwent a surgical treatment for their colorectal cancer (2). Presently, multimodal treatment is the gold standard, which generally includes some surgical approach. Owing to better outcomes from treatments available, and also to earlier stage at diagnosis, these patients have increased survival.

According to this, colorectal cancer treatment's aim is focused not only on disease control, but also on minimizing treatments’ side effects.

Pelvic surgery is one of the most common causes of urinary and sexual dysfunction, both in men and women. Every patient undergoing a surgical excision of rectal cancer (whether an abdominoperineal resection (APR) or a rectal anterior resection (RAR)) will be at risk for these side effects.

Interestingly, 20-40% of these patients do not restart sexual life after surgery and 23-69% of men identify a *de novo* sexual dysfunction (3-5). Neuronal injury is the classic explanation for the observed dysfunction. However, other factors like altered body image related to intestinal stoma or some side effects related to chemotherapy and radiotherapy may also play a role (6).

During many years, the surgical technique described by Miles (7) (abdominoperineal resection) has been the only option available for treating these patients. It is reported to be related to 44% of post-operative erectile dysfunction (8). More recently, with the development of new surgical techniques and new medical devices, the morbidity related to colorectal cancer surgical treatment has significantly decreased, mainly because of the adoption of RAR to excise upper two-thirds of rectum. It has been with the concept presented by Heald and Ryall (9), the total mesorectal excision (TME), that an improvement in disease-free survival rate and in urinary and sexual dysfunction has been observed.

The autonomic nervous system plays a major role in sexual and urinary function. Sympathetic system, through the superior hypogastric plexus and the hypogastric nerves that runs para-aortic and on the posterolateral mesorectal surface, is associated to ejaculation and urinary continence mechanism. Parasympathetic system, through the inferior hypogastric plexus, the pelvic and cavernous nerves, which are located laterally in pelvis at the level of the rectal lower third, is involved in the entire erection and micturition mechanisms. According to this, all surgical procedures in pelvis are related to some degree of dysfunction of these systems.

Some surgical techniques with nervous preservation have been proposed with the aim at decreasing these effects. Walsh and Mostwin (10), in 1984, have described the preservation of cavernous nerves in radical prostatectomy, which have accomplished an increase in the erectile function of these patients. However, this principle is still seldom applied and studied in other pelvic surgeries. On the other hand, there are some cases where there are some factors that do not allow the application of that preservation, like a locally advanced tumor or some anatomic individual variability.

Besides the clear evidence of real negative impact of these surgical techniques (whether APR or RAR) in sexual and urinary function, only a few articles have studied this subject. It seems that APR is significantly associated with higher sexual dysfunction rates than RAR. In a prospective study, Sendur et al. (11) presented a post-operative erectile dysfunction rate after colorectal neoplastic surgery of 83%. Risk factors already described are increased age, presence of stoma, radiotherapy, technique applied (11), tumor stage and surgeon's experience (12).

This study aims to evaluate sexual dysfunction after surgical treatment for rectal cancer, and to identify which risk factors may predict this side effect after surgery.

## MATERIALS AND METHODS

An observational retrospective study was performed including all male patients who underwent a surgical treatment for colorectal cancer between January 2011 and December 2014 (n=43) in Centro Hospitalar de Vila Nova de Gaia/Espinho. Data on demography, tumor location and stage, surgical technique, comorbidities and post-operative complications were collected from their medical records.

Afterwards, an inquiry questionnaire was made to every patient about its sexual habits and level of function before and after surgery, using the International Index of Erectile Function in its short form (IIEF-5). All questionnaires were applied by the same operator (follow-up of 21±12 months).

Inclusion criteria were male gender, surgical technique (APR or RAR), sexually active before surgery and with cognitive ability to answer the questionnaire. Emergent procedure, terminal state and death at the time of this study were the exclusion criteria.

A descriptive analysis was performed, defining population demographics and comorbidities. ED was defined as an IIEF-5 score lower than 22.

A comparative analysis was also performed between patients with and without erectile dysfunction (ED) after the surgery. A sub-analysis focused on patients without pre-operative ED (n=23) was made to compare those who did not experience any decrease in their sexual function with the ones who developed a *de novo* ED.

Continuous data were described using mean (standard deviation), and categorical data were expressed as numbers (frequencies). Categorical data were compared using Pearson χ2 test and continuous variables with Student t-test. A matched-pairs analysis was conducted to evaluate possible differences between the pre- and post-operative data, by means of the McNemar test. Odds ratio, adjusted to a multivariate analysis, explored associations between patient's characteristics and post-operative ED.

A two-sided p value <0.05 was considered as statistically significant. Statistical calculations were performed using SPSS^®^, version 23.0 (SPSS Inc., Chicago, IL, USA).

## RESULTS

Between January 2011 and December 2014, 122 male patients with rectal cancer were submitted to surgical treatment. A total of 79 patients were excluded: trans-anal excision (4 patients), surgery in emergency setting (2 patients), cognitive status disturbances (dementia, psychiatric disease, end-stage disease) precluding to answer the questionnaire (11 patients), sexually inactive (14 patients), not willing to participate in the study (6 patients) and deceased patients (42 patients). The remaining 43 patients were included in this study. The demographics and baseline characteristics of these patients are resumed in Table-1.

**Table 1:** Baseline characteristics of patients included in the sample.

Age (years), mean±SD		64±10
BMI (kg/m^2^), mean±SD		27±4
**Comorbidities, %**		
	Type 2 DM	33
	Smoker	33
Follow-up(months), mean±SD		21±12
**Surgical technique, %**		
	RAR	56
	APR	44
**Surgical approach, %**		
	Classic	67
	Laparoscopic	33
**Tumor location, %**		
	Upper third	33
	Middle third	34
	Lower third	33
**Tumor stage (AJCC), %**		
	II	23
	III	72
	IV	5
Length of stay (days), mean±SD		10±7
**Neoadjuvancy, %**		
	CT + RT	44
	RT alone	5
**Adjuvancy, %**		
	CT + RT	19
	CT alone	65
**Colostomy, %**		51
**Complications (Clavien-Dindo ≥ III), %**		9

**SD =** standard deviation; **BMI =** Body Mass Index; **DM =** Diabetes Mellitus; **RAR =** Rectal anterior resection; **ARP**=Abdominoperineal resection; **AJCC =** American Joint Committee on Cancer; **CT =** Chemotherapy; **RT =** Radiotherapy

The surgical procedure included a rectum anterior resection (RAR) in 22 patients (56°/o-17 with open approach, and 5 with laparoscopic approach) and an abdominoperineal resection (APR) in 19 (44%, 10 open and 9 laparoscopic). Tumor location was in the upper third of the rectum (defined as 10-15cm from anal margin) in 33%, in the middle third (5-10cm from middle margin) in 34% and in the lower third (<5cm from anal margin) in 33%. Most cases were at stage III (72%), with 23% at stage II and only 5% at stage IV. A multimodal approach was common with a neoadjuvant treatment being offered to 49% of patients (44% with radiotherapy plus chemotherapy approach and 5% with only chemotherapy). The average time interval between the end of these treatments and the surgery was 7.5 (±1.4) weeks. Adjuvant treatment was applied to 84% of patients (65% with only chemotherapy and 19% with radiotherapy plus chemotherapy). About half of the patients ended with a stoma (46% permanently and 5% temporarily). An early second surgery owing to post-operative complications was needed in 4 (9%) patients. The average follow-up time was 21 (±12) months.

The importance of sexual life for each patient can be seen in Table-2, with 7% considering it “without importance", 12% “with little importance", 15% “important", 34% “with much importance" and 32% considered it “the most important". Almost two thirds of the patients maintained an active sexual life after surgery. In 71% of patients some degree of change in sexual function has been observed, with 62% reporting some erectile impairment and 24% some ejaculatory impairment. Stoma presence have been described as a limiting factor by 39% of patients.

**Table 2:** Patients’ perception about importance of sexual activity and changes of sexual function after surgery - results of a self-evaluation tool.

**Importance of sexual activity, %**		
	Not important	7
	With little importance	12
	Important	15
	With much importance	34
	The most important	32
Post-operative sexually active patients, %		63
IIEF-5 pre-op		19.5±5.8
IIEF-5 post-op		12.3±6.8
**Noticed post-operative change, %**		**71**
	ED alone	43
	EjD alone	7
	ED+EjD	17
	Lower SD alone	2
	ED+Lower SD	2
Global ED		62
Colostomy (experienced as a limiting factor)^a^, %		39

**IIEF =** Internation Index of Erectile Function; **ED =** Erectile dysfunction; **EjD =** Ejaculatory dysfunction; **SD =** Sexual drive.

a *n*=18

**Figure 1:**
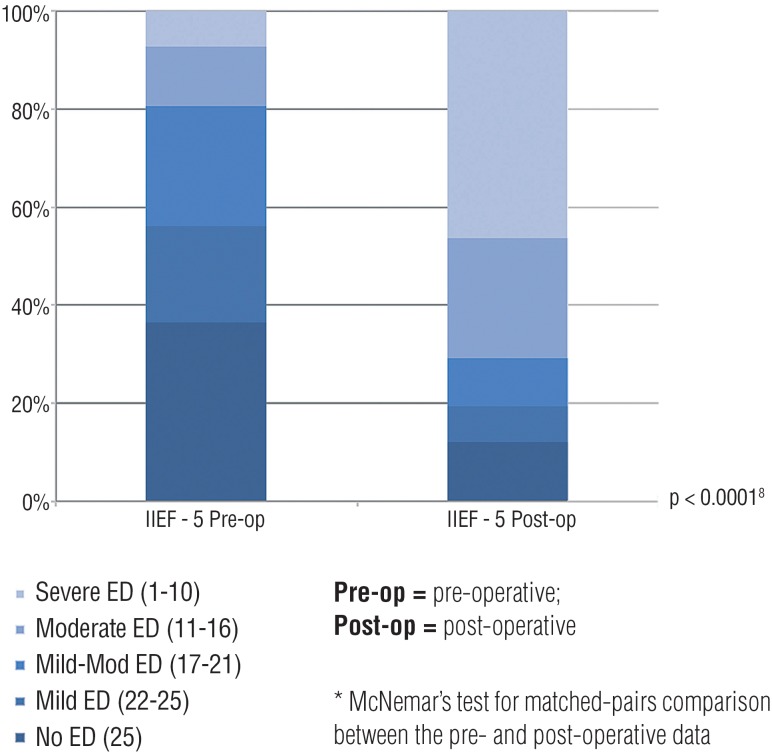
self-assessment of erectile function - comparison between pre and post-operative

The evaluation of pre-operative sexual function (Figure-1) detected that 44% of patients already had some symptoms (average IIEF-5 19.54±5.8). However, after surgery, that percentage increased to 78% (average IIEF-5 12.27±6.8, p=0.007). Data reporting ejaculatory function has been excluded since many patients were not able or found it very hard to answer to those questions (mainly those with ED).

Univariate analysis (Table-3) compared patients with and without ED after surgery and found differences in these groups regarding their age (p=0.007), surgery performed (p=0.03) and the presence of a stoma (p=0.03). A multivariate analysis (model including age, surgery performed, presence of stoma) was then performed in order to identify those factors that independently influenced the presence of post-operative ED and it revealed that only age was a predicting factor for it, with an OR of 1.145 (p=0.008).

**Table 3:** Univariate and multivariate* analysis comparing characteristics of patients with and without post-operative erectile dysfunction

		Post-operative ED (n=33)	Without post-operative ED (n=10)	
Age (years), mean±SD		66.3±9.5	57.2±6.6	***p*=0.007** ***p*** * **=0.008**
BMI (kg/m^2^), mean±SD		27.2±4.3	26.1±3.9	N.S.
**Surgery, %**				
	RAR	55	10	***p*=0.026**
	APR	45	90	p*=0.136
**Tumor location, %**				
	Upper third	30	40	
	Middle third	34	40	N.S.
	Lower third	36	20	
**Technique, %**				
	Open	70	60	N.S.
	Laparoscopic	30	40	
Length of stay (days), mean±SD		10.5±7.6	9.3±7.1	N.S.
Comorbidities (DM + Smoker), %		79	70	N.S.
RT, %		66	60	N.S.
CT, %		81	90	N.S.
Colostomy, %		60	20	***p*=0.034** p*=0.527
Complications (Clavien-Dindo>III), %		6	20	N.S.
Consider sexual life as important, %		75	100	N.S.
Follow-up (months), mean±SD		23±11	15±11	N.S.

**ED =** Erectile dysfunction; **SD =** standard deviation; **OR =** Odds ratio; **CI =** confidence interval; **BMI =** Body mass índex; **RAR =** Rectum anterior resection; **APR =** Abdominoperineal resection; **N.S =** Non-significant; **DM =** Diabetes mellitus; **RT =** Radiotherapy; **CT =** Chemotherapy

* Multivariate analysis (logistic regression including age, surgery and colostomy)

Another analysis (Table-4) focused in those patients without pre-operative ED (n=23) was performed, in order to characterize the variables associated with the development of *de novo* postoperative ED. It revealed that APR (p=0.04), lower third tumor's location (p=0.03) and presence of comorbidities (p=0.013) (namely, smokers and diabetic patients) were predictors of *de novo* ED after surgery. Another multivariate analysis (model including surgery performed, tumor's level, presence of comorbidities, presence of colostomy) was then performed in order to identify those factors that independently influenced the presence of *de novo* ED and it revealed that only the presence of comorbidities was a predicting factor for it, with an OR of 21.93 (p=0.046).

**Table 4:** Univariate and multivariate* analysis including only patients without pre-operative erectile dysfunction(ED) - comparison of characteristics of patients with *de novo* post-operative ED and patients without ED

		Post-operative *de novo* ED (n=14)	Without post-operative ED (n=9)	
Age (years), mean±SD		61.1±11.0	57.2±6.6	N.S.
BMI (kg/m^2^), mean±SD		25.7±3.6	26.1±3.9	N.S.
**Surgery, %**				
	RAR	43	11	***p*=0.040**
	APR	57	89	p*= 0.98
**Tumor location, %**				
	Upper third	7	44	***p*=0.030**
	Middle third	36	33	p*=0.452
	Lower third	57	22	
**Technique, %**				
	Open	64	56	N.S.
	Laparoscopic	36	44	
Length of stay (days), mean±SD		11.4±11.4	9.5±7.5	N.S.
Comorbidities (DM + Smoker), %		78	21	***p***=**0.013** ***p*** * **=0.046**
RT, %		64	56	N.S.
CT, %		86	89	N.S.
Colostomy, %		57	22	p=0.197 p*=0.98
Complications (Clavien-Dindo>III), %		22	14	N.S.
Consider sexual life as important, %		93	100	N.S.
Follow-up (months), mean±SD		22+13	15+11	N.S.

**ED =** Erectile dysfunction; **SD =** standard deviation; **OR =** Odds ratio; **CI =** confidence interval; **BMI =** Body mass index; **RAR =** Rectum anterior resection; **APR =** Abdominoperineal resection; **N.S =** Non-significant; **DM =** Diabetes mellitus; **RT =** Radiotherapy; **CT =** Chemotherapy

* Multivariate analysis (logistic regression including surgery, tumor location and presence of colostomy)

## DISCUSSION

This study presents the impact of rectal surgery for oncologic purposes on sexual function of male patients. In fact, 71% experienced some difference in their sexual habits. Probably the most alarming result is that 37% of patients did not resumed their sexual life.

Oncologic treatment outcomes usually consider only mortality rates or disease-free survival. However, owing to the success of these treatments, nowadays an investment on patient's quality of life is observed. It is precisely in that setting that sexuality is considered to be a major topic regarding an evaluation on someone's quality of life (13). The results that are presented support this thesis, since 81% of patients consider sexual life as being “important”, “with much importance” or “the most important”.

Amongst the dysfunctions that were found, erectile dysfunction (ED) represented the most common one. Considering the average age of this group of patients (64 years), the pre-surgical rate of ED (45%) is considered normal. Nonetheless, the increase in this rate after surgery is notorious - approximately 80%. The cause of this dysfunction is considered to be multifactorial, since factors like altered body image perception (mainly in patients with a stoma) (14, 15) and radiotherapy (16) seems to have some influence. However, probably it is the neuronal injury (chiefly at the parasympathetic nervous system, at the inferior hypogastric plexus level (6, 17)) that most contributes to this impairment, revealing that the adoption of surgical techniques with vasculo-nervous preservation when feasible, may also be important to improve outcomes. In this study, increased age, surgical technique and the presence of a stoma revealed some degree of association with the presence of ED post-operatively. Interestingly, only 49% consider the stoma to be an important factor. When trying to define the weight of each variable for the outcome of “post-operative ED” it was found that age was the only independent factor, with an estimate doubling risk of developing ED after surgery for each decade of age.

Since this analysis could be biased by a high percentage of patients already with ED in the preoperative setting, a secondary analysis managed to define risk factors for developing an ED secondary to surgery. It was found that those risk factors were the type of surgery, the level of tumor in the rectum and the presence of comorbidities. The type of surgery itself was already predictable to be a risk factor. Also the tumor level, since it influences directly the surgery approach, coincident to what other groups revealed (11). A difference in these results is mainly the importance of comorbidities for the outcome of this surgery. In the multivariate model it became stressed that type 2 Diabetes Mellitus and/or tobacco smoker, when present, increased 21 times the risk of a patient developing ED.

Another remarkable finding is that the surgical approach (open or laparoscopic) and the neoadjuvant or adjuvant therapies have not shown any association with a *de novo* ED, in this study. Minimally invasive techniques (both laparoscopic or robotic-assisted) seem promising, however additional studies are lacking to demonstrate any significant benefit towards a better sexual performance after surgery.

These results are similar to others presented by other groups, but to our knowledge, this is the first that shows a strong relationship between diabetes or tobacco abuse and the development of *de novo* ED after surgery.

This study, however, has many limitations. First, the small number of cases - similar to other series published, this is an issue that limits the statistical power of the conclusions. The absence of an independent association with established risk factors for *de novo* ED, mainly the tumor location (lower third), the APR technique and radiotherapy is elucidative of this issue. Larger studies are lacking in order to confirm results purposed by the different studies available. Second, the study design - as a retrospective study it is contaminated with many bias, like selection bias (since only patients that survived at the time of the study were included) and it does not allow to generalize these conclusions to all patients that went through these surgical approaches. Third, sexual function whether pre- or post-operative may not be completely trustworthy, since it was reported retrospectively by patients, and at different post-operative time.

The number of articles on this topic is increasing recently, however there is a long way to be traveled in order to achieve proper and definitive conclusions. Ideally, a multicenter randomized prospective study with a larger sample would answer many questions raised by many groups studying this topic, chiefly which would be the risk factors that highly increased the rate of *de novo* ED.

The simple detection of this impairment does not imply an increase in the actual quality of life of these patients. One of the major goals of this study was to try to define which patients would be at the higher risk to develop a *de novo* ED. Every patient must be aware of the risks and consequences of each procedure. However, since usually the sexual life is considered to be a sensible topic, it is expected to be the doctor to ask the patient about this domain instead of passively waiting for the patient to expose their complains. As a matter of fact, the correct identification of a patient with sexual dysfunction allows the physician to correctly direct him to proper treatment or even an evaluation on a Urology / Andrology consultation. The neuronal injury mechanism previously exposed explains the partial inefficacy of oral 5-phosphodiesterase inhibitors. Regarding studies on patients after a radical cystoprostatectomy or a radical prostatectomy, the adoption of “penile rehabilitation” before and after surgery may increase the sexual function indexes of these patients (18, 19) and might play a role in patients submitted to rectal cancer surgeries.

## CONCLUSIONS

Sexual dysfunction represents a common side effect in patients submitted to colorectal cancer surgery. Besides the high prevalence of previous sexual impairment related to the age of these patients, the negative impact in sexual function of this surgical treatment was clearly demonstrated in the present study.

The RAR approach to excise rectal tumors from the lower third (in comparison to ARP) has significantly improved morbidity outcomes in these patients, as shown in this study. The presence of comorbidities (type 2 diabetes mellitus and smoker patients) was an independent risk factor for the development of *de novo* post-operative ED.

It becomes essential to correctly evaluate and identify these patients post-operatively in order to offer an adequate therapeutic approach.

## ETHICAL STATEMENT

All subjects gave their informed consent for inclusion before they participated in the study. The study was conducted in accordance with the Declaration of Helsinki, and the protocol was approved by the Ethics Committee of Centro Hospitalar de Vila Nova de Gaia/Espinho.
